# Antibacterial Materials: Influence of the Type and Conditions of Biological Tests on the Measured Antibacterial Activity

**DOI:** 10.1002/marc.202400378

**Published:** 2024-10-22

**Authors:** Baptiste Caron, Marc Maresca, Amelie Leroux, Marie Lemesle, Jean‐Louis Coussegal, Yohann Guillaneuf, Catherine Lefay

**Affiliations:** ^1^ Aix‐Marseille Université CNRS Institut de Chimie Radicalaire UMR 7273 Marseille 13397 France; ^2^ Aix Marseille Univ CNRS Centrale Marseille, iSm2 Marseille 13397 France; ^3^ VYGON Ecouen 95440 France

**Keywords:** antibacterial surface, antibacterial test, CERTIKA, ISO 22196, ionic strength

## Abstract

In recent years, the growing problem of antibiotic resistance has highlighted the need for antibacterial materials to prevent the development of infections. Different types of tests exist to certify the antibacterial properties of materials. Variations in results can occur due to the unique requirements of each test technique. The antibacterial test result may be influenced, in particular, by the distinct modes of action of leaching and non‐leaching compounds. Using antibacterial materials prepared by the dispersion of an amphiphilic cationic synthetic copolymer in a polyurethane matrix, the influence of the reaction medium and the contact time on the results obtained by two well‐established tests: ISO 22196 and CERTIKA is investigated. This shows that the kinetics of killing is bacteria dependent and depending on the test conditions (concentration of salt, time of contact, or media), contradictory results could be obtained. Moreover, the influence of the ionic strength (called salt effect) in both free solution and antibacterial surface is highlighted.

## Graphical abstract

The impact of reaction medium and contact time is examined on the outcomes of two widely recognized material antibacterial tests: ISO 22196 and CERTIKA, using antibacterial materials made by dispersing an amphiphilic cationic copolymer in a polyurethane matrix. The rate of killing is proved to be bacteria‐dependent, meaning that varying outcomes may be achieved based on external factors (salt content, contact time, and media).

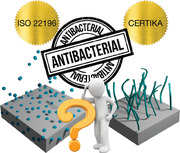

## 1 Introduction

**Figure 1:**
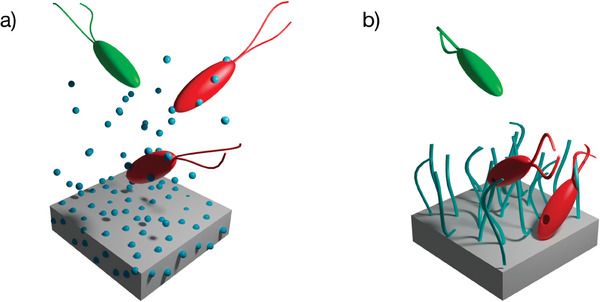
Schematic representation of antibacterial materials with a) leaching of small molecules (silver, antibiotics, biocides, etc.) and b) non leaching materials with mainly grafting of hydrophilic/ionic polymers/peptides.

Microbe adherence to surfaces has led to significant issues for health and the environment (surface contamination and bacterial proliferation, particularly in hospitals).^[^ ^1^, ^2^ ^]^ The most widely employed antimicrobial strategy involves embedding substances or reagents possessing biocidal qualities to a material and achieving antimicrobial activity by releasing biocide. Such an approach has nevertheless some drawbacks since the environment, including sometimes water, is contaminated by the biocide discharged. Release of chlorhexidine is for example considered as responsible for anaphylaxis.^[^ ^3^, ^4^ ^]^ Antibiotic resistance has also been observed after bacteria were exposed to subinhibitory concentrations of antibiotics.^[^ ^5^, ^6^, ^7^ ^]^ In addition, because of the release of the active compound, leaching materials cannot be used for a long period because of a loss of activity with time. In the case of other structures called contact‐killing materials, biocide is covalently or firmly immobilized on top of the surface.^[^ ^8^, ^9^, ^10^, ^11^ ^]^ The killing of bacteria thus occurs when microorganisms come into contact with the surface,^[^ ^12^ ^]^ limiting biocide leakage to the environment in comparison to the biocide‐releasing procedure (**Figure** **1**b).

Regulatory agencies require expensive, frequently statistically impossible, large clinical trials before allowing market introduction, and the industry demands simple, reliable, and inexpensive manufacturing processes for antimicrobial surface designs.^[^ ^13^ ^]^ As a result, many experimental antimicrobial surface designs for biomaterials or coatings reported in the literature never make it to clinical use.^[^ ^13^ ^]^ Standardized test methods are thus a necessary and important step in the development of a novel antimicrobial material. However, the established in vitro test methodologies to validate antimicrobial activity are equally as different as antimicrobial materials and additives available on the market.^[^ ^14^, ^15^ ^]^ Based on the mechanism of action of the object to be tested, these tests could be categorized into five groups: high surface‐to‐volume ratio tests, agar inhibition zone tests, suspension tests, adhesion tests, and biofilm tests. For release‐killing materials, agar inhibition zone tests, suspension tests, and high surface‐to‐volume ratio tests could be used whereas for contact‐killing materials, biofilm tests and again high surface‐to‐volume ratio tests seem more adapted. For non‐porous surfaces, and using samples with high surface‐to‐volume ratios, the ISO 22196/JISZ 2801 is the most well‐known test method in the industry. Another approach for testing non‐porous surfaces is the newly published ISO 7581:2023 consisting of testing dry bacteria deposited via aerosol spreading.^[^ ^16^ ^]^ For objects that cannot be analyzed by ISO 22196/JISZ 2801, suspension methods such as ASTM E 2149 were developed and were at the beginning recommended for contact‐killing materials but it was later realized that due to difficult transport of the bacteria to the surface, this kind of test is more compatible with release‐killing materials.^[^ ^15^ ^]^ Moreover, certain tests are more recommended in some sectors such as textiles^[^ ^17^ ^]^ or food packaging.^[^ ^18^ ^]^ For medical devices, the complete characterization of the materials should follow the ISO 10993 standard, but there is no formal regulation related to the infection risk and thus recommended tests to assess the antibacterial ability of such materials.^[^ ^13^ ^]^ The experimental conditions (bacteria strains utilized for testing, the quantity of bacteria used to inoculate the test samples, the volume of the bacterial inoculum, the length of the incubation period, the temperature, and the humidity, etc.)^[^ ^15^ ^]^ but also the shape/size of the tested product, however, vary greatly throughout the various approaches. Because every test technique has its own requirements, different results may be obtained.^[^ ^19^, ^20^, ^21^, ^22^, ^23^, ^24^ ^]^ In particular, the different modes of action between leaching and non‐leaching materials could influence the result of the antibacterial test.

To counteract this problem, Agarwal and coworkers^[^ ^25^ ^]^ proposed to use assays based on proliferation, such as the CERTIKA^[^ ^26^ ^]^ test to determine the antibacterial efficiency of medical devices. This protocol is supposed to assess the antimicrobial activity of both materials of any shape/size containing leaching and non‐leaching additives even if suspension methods were reported to be less efficient due to the difficult contact between bacterial and the surface. The special feature of this test is that it measures the division and release of daughter cells during a 18 h period following inoculation. The release of essential daughter cells, which are responsible for the development of the infection, is determined regardless of the antimicrobial mode of action. Then, over time, the growth activity of these descendent bacteria is observed and quantified.^[^ ^25^, ^26^ ^]^ Another specificity of the CERTIKA test is to requires only 1h of time contact in phosphate‐buffered saline (PBS) whereas the ISO 22196 test uses 24h in a mixture of water and nutrient broth (NB) diluted at 1/500 called water/(NB1/500).

**Figure 2:**
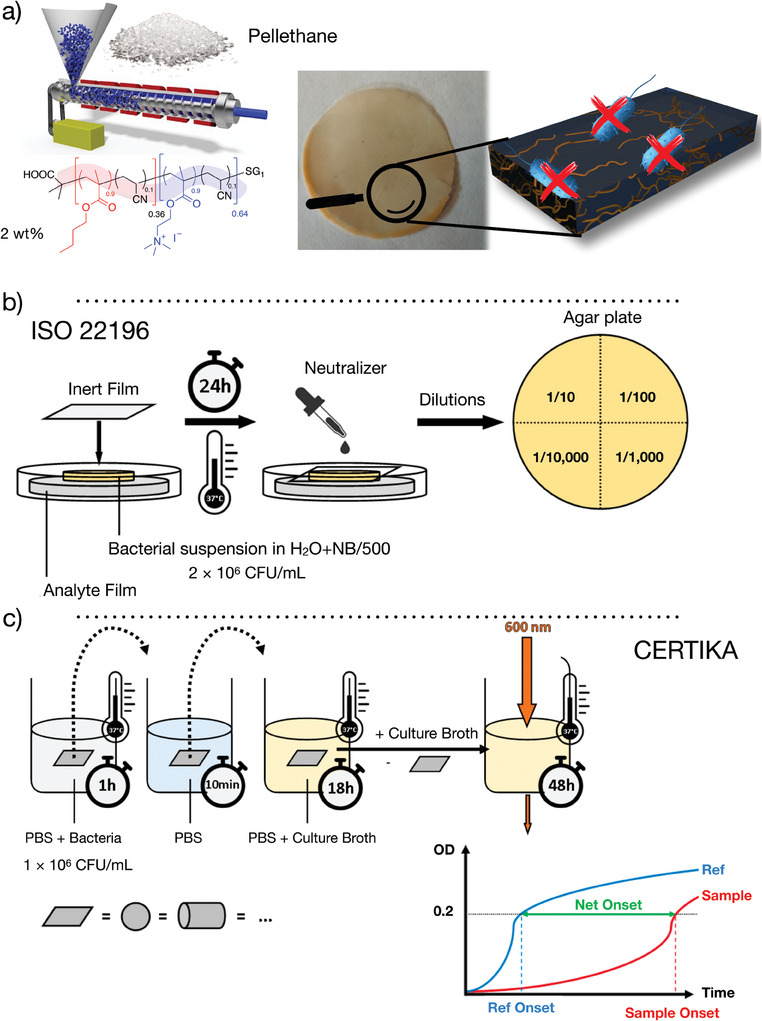
Preparation of an antibacterial organic material by the dispersion of 2 wt.% of amphiphilic cationic copolymer into the Pellethane® (PU) matrix. b) Schematic illustration of the ISO 22196 test: colony count of bacteria surviving after contact with films under incubation at 37°C for 24 h from 112 µL of bacterial suspension at 2 × 10^6^ CFU/mL in H2O + NB/500 (Results corresponds to means ± S.D. (n = 5)). c) Schematic illustration of the CERTIKA test kinetic study of the growth of bacteria adhered to analyte surfaces after incubation at 37°C for 1h from a bacterial suspension at 1 × 10^6^ Colony Forming Units (CFU)/mL in PBS. Optical density measurements were taken every 30 min for 48 h using absorbance at 600 nm at 37°C. (Results correspond to means (n = 8)).

Recently, we developed an easy and straightforward method to turn non‐active organic material into equivalent but non‐leaching antibacterial plastics. More precisely we have shown that synthetic amphiphilic cationic antimicrobial diblock copolymers based on methacrylic monomers are effective antibacterial compounds that can be used as additives for common organic materials such as PETG, PE, and PLA.^[^ ^27^, ^28^, ^29^ ^]^ The use of less than 2 wt.% of copolymers that are melt‐blended with pristine organic matrix prior to the preparation by extrusion of solid organic materials conferred antibacterial activity against Gram+ (*S. aureus*) and Gram‐ (*E. coli*) model bacteria (**Figure** **2**a).^[^ ^27^, ^28^, ^29^ ^]^ In the previous studies, we used a modified ISO 22196 antibacterial test and in this new study, we investigated the use of the CERTIKA test and tried to highlight its advantages and limitations (Figure 2b,2c).

## 2 Results and Discussion

### 2.1 Preparation of the Antibacterial Film

Since one of the most common applications for antibacterial materials is in the biomedical field, we first turned our attention to polyurethane (PU) matrix and more precisely to Pellethane®. We thus prepared some antibacterial pellethane‐based film by blending 2 wt.% of our antibacterial copolymer with the virgin pellethane matrix in an internal mixer.^[^ ^28^, ^29^ ^]^ The copolymer we used as an additive is a poly(butyl methacrylate)‐*b*‐poly(dimethylaminoethyl methacrylate) (PPBMA‐*b*‐PDMAEMA) diblock copolymer of ≈20 kDa, and 70 mol.% of DMAEMA that has been post‐polymerization quaternized with methyl iodide (MeI). Such a high molecular weight prevents any eventual leaching of the additive out of the PU matrix to obtain non‐leaching antibacterial PU.

**Figure 3:**
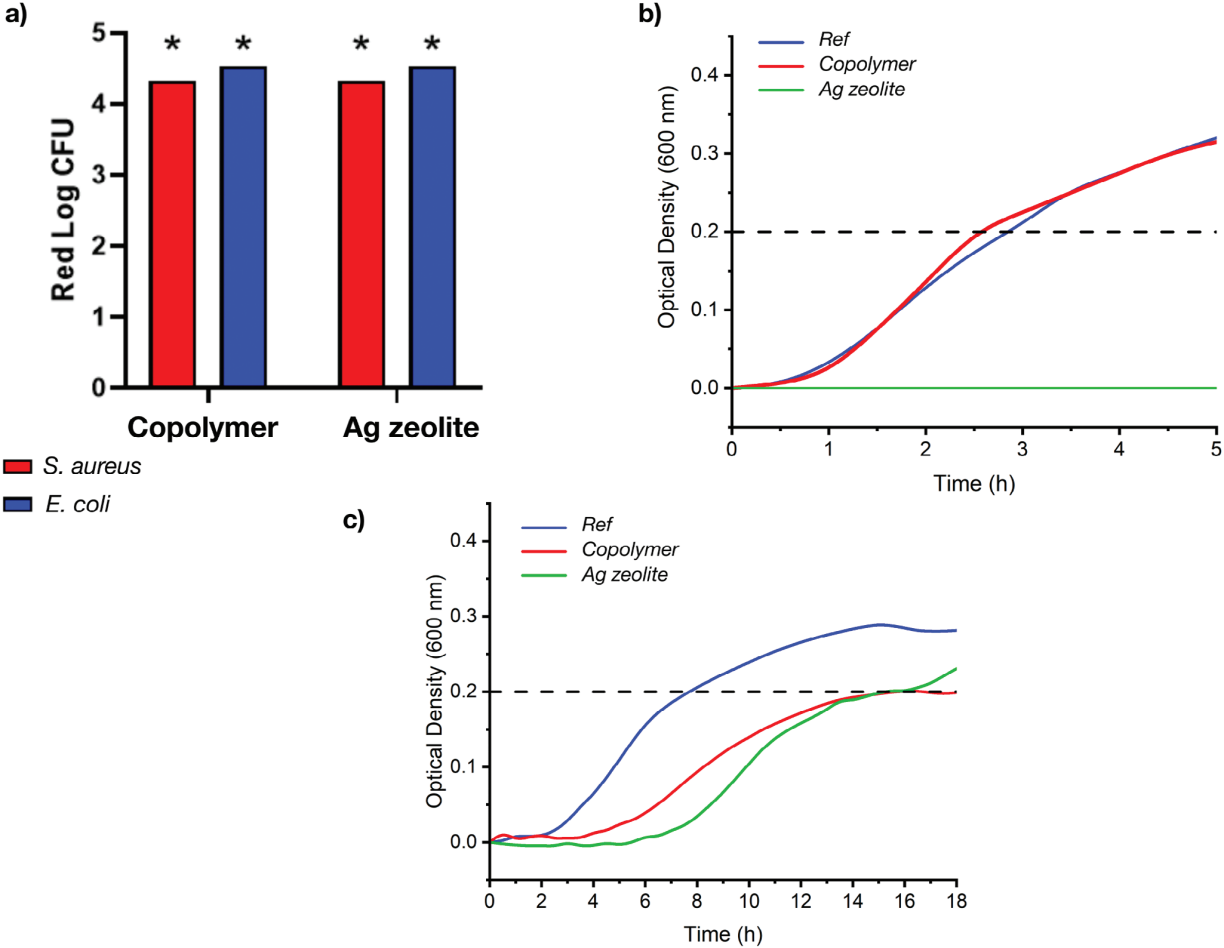
a) Bactericidal activity (ISO 22196 test) of the PU films with silver zeolite or with 2 wt.% of antibacterial copolymer on E. coli and S. aureus. The results correspond to means ± S.D. (n = 3), ^*^ corresponds to 100% killing. b) CERTIKA Test for the PU films with silver zeolite or with 2 wt.% of antibacterial copolymer on E. coli. Evolution of the daughter bacterial growth over a period of 48 h. c) CERTIKA Test for the PU films with silver zeolite or with 2 wt.% of antibacterial copolymer on S. aureus. Evolution of the daughter bacterial growth over a period of 48 h.

The masterbatch was then extruded and films were obtained using a hot press (see experimental section for details). The PU with 2 wt.% of antibacterial copolymer was compared with two reference films: PU without any additive (noted Ref in the figures) and an antibacterial leaching PU containing silver zeolite as an antibacterial leachable active molecule. The films were then tested according to the ISO 22196 antibacterial test on *E. coli* (ATCC 8739) and *S. aureus* (ATCC 6538P). This test was preliminary chosen since it is the most used test in the industry. Second in the case of catheter for example, it was also relevant since most of the contamination of the devices was on the external surface of it and bacteria are more in close contact between the materials and the epithelial cells than via mass transport from a liquid volume.^[^ ^13^ ^]^ In this test, a bacterial solution is deposited between the active surface and a sterile coverslip, with a contact time of 24 h in water/(NB1/500). The medium was then collected and the reduction of microorganisms relative to initial concentrations and the control surface was calculated. The results of the tests are reported in **Figure** **3**a.

For both the silver ion‐containing polyurethane matrix and the one containing the non‐leachable copolymer, a complete killing of both Gram‐ and Gram + bacteria was observed, and the reduction logarithms (Red Log)s were above the limit of 3. This confirms our previous findings on the efficiency of our approach to prepare easily antibacterial materials by the dispersion of an amphiphilic cationic copolymer as an additive.^[^ ^28^, ^29^ ^]^

After this ISO 22196 test, we then focused on the CERTIKA test which was supposed to be more universal since it relies on the evolution of daughter bacteria and could be used with samples of any shape and mode of action.^[^ ^25^, ^26^ ^]^ This test is also possible for other samples than a film and thus was practically relevant for commercially‐available devices where no planar surfaces could be tested. To compare the two techniques, we then used the same films in this study. This test first consists of the incubation of 1h at 37 °C of the film with a bacterial suspension in PBS. After the bacterial suspension removal, the contaminated film is let incubated for 18h at 37°C. The film is finally removed, and the medium is monitored to follow the bacterial growth. The net onset time, defined as the time difference to reach a 0.2 optical density (Figure 2), between the reference and the antibacterial film allows us to determine the efficiency of the antibacterial treatment. Similarly to the ISO 22196 test, both *E. coli* (ATCC 8739) and *S. aureus* (ATCC 6538P) were tested, and the results are presented in Figure 3b,3c respectively. In the case of the silver ion‐containing polyurethane matrix, a net onset of 7.8 h was observed for *S. aureus*, and no bacterial growth for *E. coli* was obtained. These results are in good agreement with the previous study of Agarwal and coworkers^[^ ^25^ ^]^ that observed an onset of 5 h in the case of MRSA and no growth for *E. coli*. The polyurethane film containing the copolymer presented totally different results depending on the nature of the bacteria. In the case of *E. coli*, a bacterial growth similar to the one observed on the reference film was monitored indicating no activity. In the case of *S. aureus*, a net onset of 8.6 h. Since it was shown that in this test, the bacteria divide every 30 min, the net onset could be related to a log reduction.^[^ ^25^ ^]^ An onset of 6h corresponds to a Red Log of 3 and thus the 8.6 h could be related to a Red Log of 5.2 confirming the activity of the copolymer. In the case of *E. coli*, the two tests (ISO 22196 and CERTIKA) were thus not in agreement and deeper analysis is mandatory to conclude on the efficiency of the films containing the copolymer.

### 2.2 Influence of the Salt Concentration in the Test Medium

In order to understand this peculiar behavior, a closer look at the two procedures highlights the difference in incubation medium between these two tests. The ISO 22196 test used a water/(NB1/500) medium whereas the CERTIKA test requires the use of PBS. The main difference between these 2 media is the concentration of NaCl (8g/L) in the PBS instead of 10 mg L^−1^ for water/(NB1/500). It is known that antimicrobial peptides and their synthetic analogs, i.e. cationic amphiphilic copolymers could be sensitive to the salinity of the medium.^[^ ^30^, ^31^, ^32^ ^]^ Such sensitivity is not fully understood but could be linked to the polyelectrolyte effect that modifies the conformation of the charged macromolecules in the presence of free salts^[^ ^33^ ^]^ and then modifies their interaction with the bacterial membrane. Such a membrane could also be impacted by a higher concentration of salt. To first test if the copolymer itself is salt‐sensitive, we determined its minimal inhibition concentration (MIC) vs *S. aureus* and *E. coli* in both water/(NB1/500) with and without NaCl at 8 g L^−1^ and also in a solution of PBS containing MH broth (prepared by dilution a PBS10x solution by MH). The results are presented in **Table** **1**. The results first showed that in water/(NB1/500) the copolymer is far more efficient on *S. aureus* than *E. Coli*. This result is expected since *E. coli* is a Gram‐ bacteria whose sensitivity to synthetic antimicrobial polymer is reported to be lower due to the presence of a double membrane structure.^[^ ^34^, ^35^ ^]^ The impact of the medium and especially of the salt concentration on the efficiency of antibacterial copolymers is not widely reported but in some studies, the introduction of salt impacted drastically the results.^[^ ^34^, ^36^, ^37^ ^]^ With this copolymer, the MIC value of the polymer prepared in PBS is similar in the case of *S. aureus* but increased up to 400 µg mL^−1^ in the case of *E. coli*, confirming the reduced efficiency of the copolymer with *E. coli* or maybe also the higher sensitivity of the *E. coli* membrane to the ionic strength of the external media.

**Table 1:** Minimal inhibitory concentration (MIC) of the antibacterial copolymer against *E. coli* and *S. aureus* in function of the quaternary ammonium's counter‐ion and media (measured in Mueller–Hinton broth (MH) with or without 8 g L^−1^ NaCl; or a solution prepared with 1vol of PBS10x diluted with 9 vol MH (PBS‐MH)).

	Minimal Inhibitory Concentration (MIC) (µg/mL)							
Bacterial strain		*E. coli*				*S. aureus*		
Medium		MH	MH + NaCl	MH‐PBS		MH	MH + NaCl	MH‐PBS
Cl^−^ Counter‐ion		100	> 400	> 400		12.5	25	50
I^−^ Counter‐ion		100	> 400	> 400		12.5	25	25

**Figure 4:**
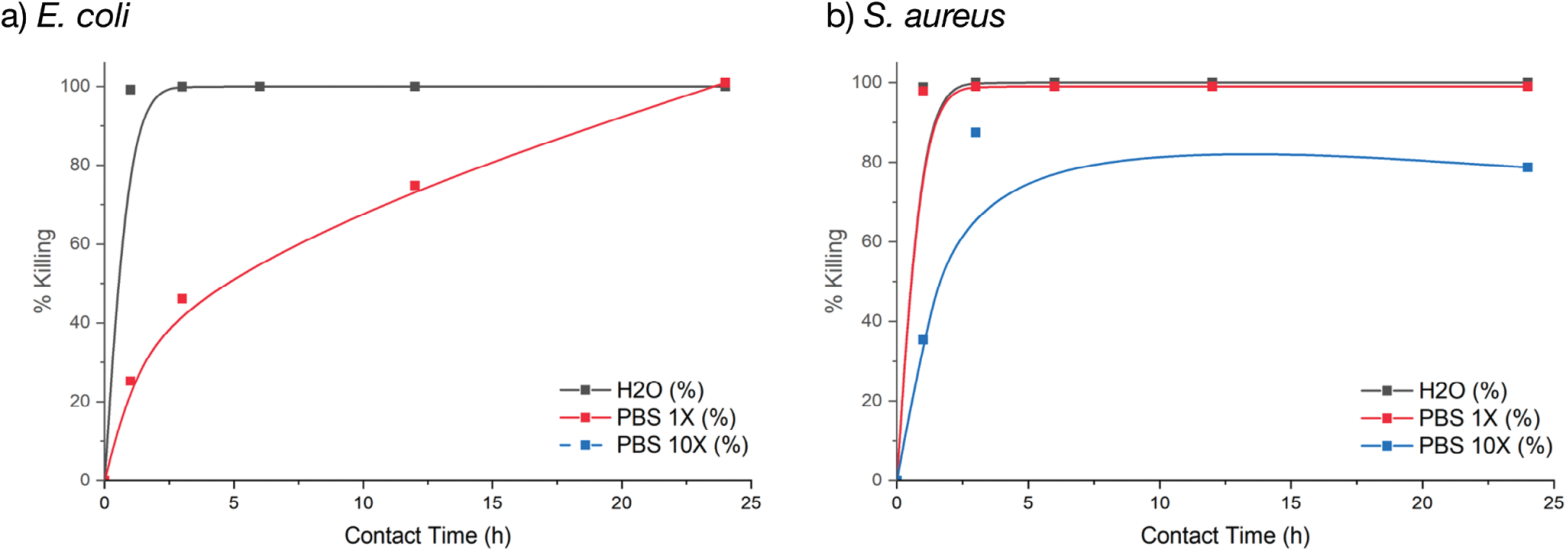
Antibacterial kinetic study (% killing) of non‐leaching polyurethane containing 2wt.% of quaternized PBMA‐*b*‐PDMAEMA copolymer against a) *E. coli* and b) *S. aureus* incubated in H_2_O or PBS media using the ISO 22196 protocol. The lines are guides for the eyes only.

The antibacterial cationic copolymers used as additives are obtained after a quaternization step using MeI. This implies that the counter ion of the copolymer is I^−^. However, since the films are immersed in PBS containing a large excess of NaCl, counterion metathesis could occur on the copolymer at the film surface^[^ ^38^ ^]^ and this could impact its antibacterial efficiency. To investigate this hypothesis, we prepared prior to the MIC determination a Cl‐based copolymer by performing the counterion metathesis using an ion‐exchange resin. The results presented in Table 1 do not show any difference, ruling out the impact of the counterion on the bacterial efficiency at least of the copolymer in solution. Besides the effect of salt on the copolymer that is free in solution, the effect of salt onto the surface has also to be considered. It is also known that the non‐electrostatic interactions between polymers and solvents, chain entropic elasticity, and counterion‐induced osmotic pressure are the main parameters to describe the swelling behavior in films decorated with polycationic chains. In the so‐called salted regime, the polycationic chains could collapse on the film as the ionic strength of the medium increases because the difference in ion concentration within and outside the film reduces.^[^ ^39^ ^]^ To test the different hypotheses, we then performed ISO 22196 tests in both PBS and water/NB(1/500) and investigated the influence of the duration of the contact time on the efficiency of the bacterial killing. The results are presented in the **Figure** **4**.

First, the killing of the antibacterial film on *E. coli* and *S. aureus* in water/NB(1/500) which is the traditional medium of the ISO 22196 test is very quick and efficient with 100% killing in one hour, showing the versatility of the approach. The ISO 22196 test recommends 24h of contact, whereas the CERTIKA used 1 hour of contact. In the case of *S. aureus*, the addition of salt (PBS) does not impact the efficiency which is similar to the effect in solution in water/NB(1/500). In the contrary, the efficiency of the killing for the antibacterial film in the case of *E. coli* is strongly dependent on the medium with a marked slowdown of the rate of killing but the final efficiency after 24h of contact is still preserved. This result is coherent with the contradictory results obtained with the ISO 22196/CERTIKA tests presented on Figure 3 since different contact times were investigated. The good efficiency after 24h of the antibacterial materials on *E. coli* (Figure 4) but with a slower kinetics do not confirm the lower efficiency of the free copolymer in solution (Table 1, MIC > 400 µg mL^−1^) demonstrating that the two systems (free copolymer and antibacterial solid material) are different. Second, the good efficiency with both media for *S. aureus* do not allow to envision any collapse of the copolymer on the surface at such a concentration. We then extended these tests to PBS 10x to increase the ionic strength of the medium. At this salt concentration, Thormann and coworkers^[^ ^39^ ^]^ reported the collapsing of polycationic film made of *n*‐butyl methacrylate and 2‐(methacryloyloxy)ethyl]trimethylammonium chloride grafted on wafers. At such a high salt concentration, it was not possible to grow *E. coli* in this medium whereas the test was still possible for *S. aureus*. In the case of *S. aureus*, the efficiency was both slowed down and lowered (Figure 4) which is consistent with the collapsing of the cationic copolymer that was embedded onto the surface of the film.

**Figure 5:**
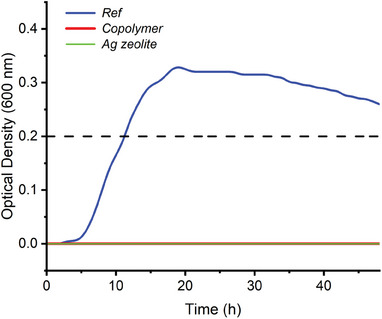
Measured antibacterial activity of non‐leaching polyurethane containing 2wt.% of quaternized PBMA‐*b*‐PDMAEMA copolymer by CERTIKA tests against *E. coli* incubated in water/NB(1/500) instead of PBS with a contact time of 1 h.

Finally, since we showed that the failure of the CERTIKA test is related to the medium and/or the contact time between the materials and bacteria, we modified the CERTIKA test by performing the test in water/NB(1/500) with still 1h of contact. The results of this modified test are presented in **Figure** 5 and all the CERTIKA results are gathered in **Table** **2**.

**Table 2:** CERTIKA test results depending on the bacteria (*E. coli* or *S. aureus*), material (non‐leaching antibacterial PU with 2 wt.% of antibacterial copolymer or Ag‐based leaching antibacterial PU), and media used (PBS or water/NB(1/500) with 1h of contact time.

Bacterial Strain		*E. coli*				*S. aureus*			
Medium / Time	PU Sample	Onset (OD = 0.2)	Net Onset	%Killing	RedLog CFU	Onset (OD = 0.2)	Net Onset	%Killing	RedLog CFU
PBS / 1 h	Ref (no additive)	2.9	‐	‐	‐	7.7	‐	‐	‐
	with 2 wt.% copolymer	2.6	0	0	0	16.3	8.6	99.99934	5.18
	with Ag Zeolite	> 10	> 7.1	> 99.99469	> 4.27	15.5	7.8	99.99799	4.70
H2O + NB1/(/500) / 1 h	Ref (no additive)	11.4	‐	‐	‐	7.8	‐	‐	‐
	with 2 wt.% copolymer	> 48	> 36.6	100	> 8	> 18	> 10,7	> 99.99996	> 6.4
	with Ag Zeolite	> 48	> 36.6	100	> 8	> 18	> 10,7	> 99.99996	> 6.4

The CERTIKA test performed against *E. coli* in water/NB(1/500) instead of PBS and keeping the classic 1 h of contact time showed a strong antibacterial effect with no noticeable growth of bacteria. This result is in total contradiction with the test performed in PBS (Figure 3), confirming the importance of the reaction medium on the efficiency of the test. This result is nevertheless in agreement with the ISO 22196 test that showed a very fast killing of the bacteria in water/NB500.

A decrease of the antibacterial activity of the Ag‐based leaching antibacterial material is also observed in the presence of 8 g L^−1^ of NaCl but in a very lower extend than in the case of the non‐leaching material. A stronger sensitivity of *E coli* than *S aureus* to the media is noted as well.

## 3 Conclusion

The growing issue of antibiotic resistance in recent years has brought attention to the necessity of antibacterial materials to stop infections from spreading. There are several available tests to assess the material's antibacterial qualities. Because every test technique has different requirements, there may be variations in the results. Moreover, the different mechanisms of action of leaching and non‐leaching active antibacterial molecules may have a special impact on the antibacterial test outcome. In the case of an Ag‐leaching antibacterial material used as a comparison, the modifications of the biological test conditions (bacteria, media, and time contact) do not seem to strongly influence the measured antibacterial efficiency of the material. On the contrary, by dispersing an amphiphilic cationic synthetic antibacterial copolymer (2 wt.% of quaternized PBMA‐*b*‐PDMAEMA) in a polyurethane matrix (Pellethane®) to create non‐leaching antibacterial materials, we showed in this study that the reaction media (and in particular the NaCl concentration) and the contact time (1 or 24h) can have a strong influence on the outcomes of two widely recognized antibacterial tests, CERTIKA and ISO 22196. We also demonstrated that the kinetics of killing are dependent on the bacteria, *S. aureus* being the more sensitive and independent of the antibacterial test whereas with *E. coli*, the two tests (ISO 22196 and CERTIKA) are contradictory. The influence of the NaCl concentration (8 g L^−1^ in PBS) in these tests is also emphasized for both antibacterial surfaces and free antibacterial copolymer solutions.

Investigation of the experimental conditions with this bacterium showed that the kinetics of killing is strongly slowed down by adding salt in the reaction medium, but the efficiency was not affected. This led us to modify the experimental conditions for the CERTIKA test. By replacing PBS with water/NB(1/500) as the reaction medium and keeping 1h of contact, the result is totally modified from zero to a perfect efficiency. These results outcome that the conclusion of the performed test is strongly dependent on the experimental conditions.

This article thus confirmed the good antibacterial efficiency of plastics having amphiphilic cationic synthetic antibacterial copolymer dispersed in the matrix with good results (beyond 3 CFU Log Red whatever the conditions) using the ISO 22196 test and an efficiency depending on the salt concentration using the CERTIKA test. As already stated from contact‐killing materials, if the copolymer approach is less efficient to prevent infections in the case of diluted bacteria solutions (due to the necessary contact between bacteria and surface), this methodology is still relevant for medical devices where bacteria are in close contact with the material.

## 4 Experimental Section

### Materials

The antibacterial copolymer was prepared according to a published procedure.^[^ ^27^, ^28^, ^29^ ^]^ Amberlite® IRA‐400 (chloride form) was purchased by Sigma‐Aldrich. Radio‐opaque Pellethane® was provided by Vygon. A masterbatch of silver zeolite containing Pellethane was also provided by Vygon. *Escherichia coli* (*E. coli*) ATCC 8739 and *Staphylococcus aureus* (*S. aureus*) ATCC 6538P were purchased by DSMZ. Sterile water (HyPure^TM^ Molecular Biology Grade) was provided by Cytiva. Dulbecco's PBS (10X, without Ca and Mg, sterile) from MP Biomedicals was diluted 10 times in sterile water to form PBS 1X. Nutrient Broth (NB), Tryptic Soy Broth (TSB), Luria Bertani (LB), Mueller Hinton (MH), and Agar were purchased by Thermo Fisher as broth powders and were solubilized at 13, 30, 25, 21, and 15 g L^−1^ respectively and sterilized by autoclaving.

### Preparation of Films Containing the Antibacterial Copolymer

The PU granules were dried at 90°C under vacuum before use. The premixes required to form homogeneous polyurethane (PU, pellethane®) matrix materials were prepared on a HAAKE Rheomix OS internal mixer using a HAAKE PolyLab OS Rheodrive 7 controller at 80 rpm and 200°C. Copolymer is added to the melted granules and blended for 2 min. Once cooled to room temperature, the mixture is immersed in a liquid nitrogen bath before being reduced in a FRITSCH Pulverisette 19 universal knife mill to obtain chips that can then be extruded.

The extrusion was performed on a twin‐screw mini‐extruder HAAKE MiniLab II. The processing temperature is set according to the characteristics of Pellethane®, *i.e*. 210°C, and the screws rotate at 80 rpm during extrusion. As the matrices are either premixed or easily homogenized, the melt polymer does not cycle in the machine and remains in the extruder for an average of 4 min. At the end of the extrusion, a rod is recovered. Films of 2 cm in diameter and 100 microns of thickness were then prepared using a hydraulic press (model AtlasTM Series Heated Platens from EuroLab) at 170°C.

### ISO 22196 Test

Reference and antibacterial films of 3 cm of diameter were sterilized (EO, UV, etc.). These samples were placed in a six‐well plate before being overlaid with a 112 µL drop of a bacterial suspension containing 2 × 10^6^ bacteria.mL^−1^ in sterile water with 0.2 wt.% NB or PBS. This drop was then flattened by a piece of Stomacher^TM^ homogenization bag cut into a 2 cm square and UV sterilized beforehand, to increase contact between the bacteria and the surface being tested. Films were then incubated at 37 °C for 24 h (modified incubation time for kinetic study) before adding 5mL of TSB o dilute the bacteria and stop the contact killing. This new mixture was added to a further 5mL of TSB (1:10 dilution), followed by further dilutions to 1:100; 1:1000, and 1:10,000 in sterile water. 10µL of each dilution was placed in a quarter of a petri dish on an agar gel and then gently spread over the entire area. After one night at 37°C, the number of colonies formed could be counted and the number of living bacteria determined. For each sample, the test was carried out in quintuplicate.

### CERTIKA Test

Pieces of 0.4 cm square analytical film were cut and sterilized for 15 min under UV light. These pieces were placed in the wells of 96‐well plates and brought into contact with 200 µL of bacterial suspension at 10^6^ bacteria. mL^−1^ in PBS or sterile water containing 0.2 wt.% NB. After the plates were incubated at 37°C for 1 h, the analytes were transferred to new wells and washed with 200 µL of PBS under orbital shaking at 400 rpm for 10 min. Analytes were then sterilely transferred back to new wells to which 200 µL of 20% NB solution in PBS were added. The new wells were incubated at 37°C for 18h before the analytes were finally removed from the solution. To each of the solutions, 50 µL of TSB was added before carrying out a kinetic study of OD changes by absorbance at 600 nm on a BioTek Synergy Mx plate reader. This kinetic study lasted 48 h and measurements were taken every 30 min, preceded by 10 s of orbital shaking to monitor the growth of the daughter bacteria.

### Ion Metathesis

5g of initial antibacterial copolymer (I^−^ counterion) were solubilized in 650 mL of deionized water before adding 16 g of Amberlite IRA‐400 Chloride form ion exchange resin under agitation at 1400 rpm for 2 days. The resulting mixture was filtered on a Büchner to recover the filtrate and the water evaporated under vacuum to recover the antibacterial copolymer with a chloride counterion.

### MIC Determination

Solutions containing 4 mg. mL^−1^ of the antibacterial copolymer were prepared in sterile water or sterile PBS. In parallel, a suspension containing 10^5^ bacteria. mL^−1^ in MH was also prepared from an overnight culture. In a 96‐well PP plate, 150 µL of bacterial suspension were poured into each well (except for the sterile reference in the last row of the plate) and 120 µL of MH were added to the last column of the plate. 30 µL of the different analytes (one per well) were added to each well in the last column. A cascade dilution was performed, diluting by two each time. The first column was kept with the bacteria alone for a bacterial growth test. Plates were incubated at 37°C for 24 h without orbital shaking. Finally, the MIC for each analyte was checked by eye by locating the well corresponding to the lowest concentration without turbidity corresponding to bacterial growth.

To complete the study of the salt effect on MIC, some assays were performed in other media. Both MH‐NaCl (8g. L^−1^) was prepared by diluting ten times a NaCl solution at 1.5 m in water in MH and MH‐PBS prepared by diluting ten times PBS 10X in MH was used as salty medium.

## Conflict of Interest

The authors declare no conflict of interest.

## Data Availability

The data that support the findings of this study are available from the corresponding author upon reasonable request.
